# Parent Programs for Reducing Adolescent’s Antisocial Behavior and Substance Use: A Randomized Controlled Trial

**DOI:** 10.1007/s10826-015-0263-y

**Published:** 2015-08-09

**Authors:** Camilla Jalling, Maria Bodin, Anders Romelsjö, Håkan Källmén, Natalie Durbeej, Anders Tengström

**Affiliations:** STAD, Stockholm Center for Psychiatry Research and Education, Karolinska Institutet, Norra Stationsgatan 69, 113 64 Stockholm, Sweden; Department of Knowledge-Based Policy and Guidance, National Board of Health and Welfare, Stockholm, Sweden; Department of Public Health Sciences, Social Medicine, Karolinska Institutet, Stockholm, Sweden; Department of Clinical Neuroscience, Stockholm Centre for Psychiatry Research and Education, Karolinska Institutet, Stockholm, Sweden; Department of Clinical Neuroscience, Stockholm Centre for Psychiatry Research and Education, Karolinska Institutet and Karolinska University Hospital, Stockholm, Sweden

**Keywords:** Antisocial behavior, Substance use, Adolescent, Parent training, RCT

## Abstract

Two theoretically based parent training programs, delivered in real-world settings by the social services, were examined in this randomized controlled trial for effectiveness in reducing adolescents’ antisocial behavior and substance use. Two hundred and thirty-seven (237) adolescents in ages between 12 and 18 and their parents were assigned to one of two programs or to a wait-list control condition. The programs were the nine weekly group sessions program Comet 12–18 (Swedish Parent Management Training Program) and the six weekly ParentSteps (Swedish shortened version by Strengthening Families Program 10–14). Outcome measures were antisocial behavior, substance use, and delinquency, and psychosocial dysfunction. Data based on adolescents’ and parents’ ratings of the adolescents’ problem behavior at baseline and 6 months later were analyzed with repeated measures ANVOA, Logistic regression, and Kruskal–Wallis *H* test. The results showed that parents’ ratings of adolescents’ antisocial behaviors decreased significantly over time, but no time by group effect emerged. No program effects were found in the adolescents’ self-reported antisocial behavior, delinquency, or psychosocial functioning. A threefold risk of illicit drug use was found in both intervention groups. The results suggest that neither Comet nor ParentSteps had beneficial effects on adolescent’s antisocial or delinquent behavior, or on alcohol use. The only significant group difference found was a threefold risk of drug use in the intervention adolescents at follow-up, but for several reasons this finding should be interpreted with caution.

*Trial registration number*: ISRCTN76141538.

## Introduction

Antisocial behavior has been broadly defined as behavior that offends other individuals, property, social norms, or rules and laws (Bloomquist and Schnell 2002; Lahey and Waldman 2005). In younger children, antisocial behavior often appears as disruptive behavior and verbal or physical aggression in daycare or school settings, while antisocial behavior in adolescents might also include delinquent behavior and substance use. Younger children with antisocial behavior are known to be at elevated risk for continued problems and youth violence (Lösel and Farrington 2012) as well as for poorer functioning in life domains such as education, employment, and personal relationships (Moffitt 1993). Against this background, early prevention has been suggested as an important policy with respect to childhood problem behavior (Welsh and Farrington 2007), and various interventions for parental support have been developed and evaluated over the last several decades. In Sweden, support for parents in their parenting roles has been a governmental priority for the last 10 years (National Institute of Public Health 2006), and a number of programs have been developed and studied on initiative from the Ministry of Health and Social Affairs, the Public Health Agency of Sweden (SOU 2008), and the National Board of Health and Welfare (2014). Parent programs are offered within the social services, but also in clinics, schools, youth clinics, and by non-governmental organizations, and they commonly aim at altering the parents’ behavior, improving their communication skills, and increasing their knowledge of how to promote their children’s prosocial behavior.

Parent management training (Kazdin 2005) is an umbrella term for parenting programs based on operant learning and social learning principles such as the Parent Management Training—Oregon Model [PMTO™; (Forgatch and Patterson 2010)] and the Incredible Years program (Webster-Stratton 1984). By teaching parents to use more positive parenting skills (e.g. praising children when engaging in prosocial behavior) and less negative and harsh parenting practices, these programs aim to support prosocial behaviors in children. A systematic review from the Cochrane Collaboration shows that group-based cognitive-behavioral parenting programs are effective for decreasing behavioral problems in younger children with clinical-level problems (Furlong et al. 2012). Typically, interventions in the 13 trials included in the Cochrane review encompassed 10–15 group sessions where brief videotaped vignettes of typical parent–child interactions were used for observation and modeling. Other typical components were behavior rehearsal through role-play, group discussions, and homework assignments. One of the studies included in this review evaluated the effects of the Swedish program Comet, which targets parents of children aged 3–11 years (Kling et al. 2010). The results from that study were consistent with the overall review findings and indicated improved parenting skills and lower problem levels in children of the parents in the Comet group compared to a wait-list control immediately post-treatment (Kling et al. 2010). However, the authors of the review emphasize that the results of the review are not necessarily generalizable to group-based cognitive-behavioral parenting programs for children of other age groups and problem levels (Furlong et al. 2012). The Comet program for parents of children 3–11 years was also found to be effective in a more recent and larger Swedish randomized trial (Stattin et al. 2015).

Parenting and family programs that primarily aim to prevent underage alcohol drinking and substance use have also been shown to be effective (Hindelang et al. 2001; Smit et al. 2008). One well-known intervention from the US is the Strengthening Families Program for Parents and Youth 10–14 (SFP 10–14) (Kumpfer et al. 1996). The SFP program was initially intended for substance-abusing parents and their children, but it has been modified over the years to meet the needs of new populations (Kumpfer et al. 1996). The SFP 10–14 for school-based implementation is offered to all families and children in a class, i.e. regardless of the children’s level of risk for developing or consolidating antisocial behavior. It is a multimodal intervention that includes separate sessions for parents and children as well as joint family sessions. The program has its theoretical basis in the Resilience Model (Richardson et al. 1990) and the Social Ecology Model of Adolescent Substance Use (Kumpfer and Turner 1990), and it primarily aims at strengthening protective factors for adolescent and parents. Each session addresses a particular protective and corresponding risk factor. All parent sessions are video-based and aim to improve, for example, communication skills, family bonding, and expectations concerning substance use (Kumpfer et al. 1996; Skärstrand et al. 2008). Results from a systematic review point out the SFP 10–14 as one of the few programs with promising longer-term outcomes when evaluated in US studies (Foxcroft et al. 2003). In 2008, the SFP 10–14 was adapted to match a Swedish context and was given the name Steg-för-steg (Step-by-Step) (Skärstrand et al. 2008). The cultural adaptation left the contents largely intact but profoundly affected the program format because the majority of joint parent–child sessions were omitted (Skärstrand et al. 2008). When later evaluated in a cluster-randomized trial encompassing 19 schools in the Stockholm area, no effects of the Step-by-Step intervention were found on youth’s drinking or substance use (Skärstrand et al. 2014). Among the possible reasons for the null findings was the omission of the joint parent–child sessions (Skärstrand et al. 2008).

Like in younger children, antisocial behavior among adolescents has been associated with low functioning in school and relationship domains, and also with a generally poor adult outcome (Brosnan and Carr 2000). In order to decrease early signs and to prevent the consolidation of antisocial behavior in older adolescents, the Comet and Step-by-Step programs described above were later adapted into versions for parents of adolescents—Comet for parents of youth 12–18 years old (Forster and Livheim 2009) and ParentSteps for parents of youth 13–17 years old (Larsson et al. 2009). Both of these programs are run at the secondary prevention level, i.e. they target parents of adolescents with early signs of antisocial behavior and who are at risk of maintaining such behavior. Each of the two programs is based on the same theories as the versions for the younger ages described above. Both programs are unimodal and involve parents only, meaning that the ParentSteps deviates from the SFP and also the Swedish Step-by-Step in this respect. Though the prevention of substance use is a more salient target domain for ParentSteps than for Comet 12–18, the programs share the general aim of preventing antisocial behavior and improving psychosocial functioning in at-risk adolescents.

While the notion of addressing antisocial behavior early in children´s lives has been supported in several reviews and studies (Furlong et al. 2012; Piquero et al. 2009; Salari et al. 2014; Webster-Stratton and Hammond 1997), less is known about the effects of parent interventions for at-risk adolescents. It is quite common for adolescents to temporarily engage in different antisocial behaviors, but it is often difficult to determine whether antisocial behavior in a specific individual is adolescence-limited or life-course persistent (Moffitt 1993). Addressing at-risk adolescents in order to decrease early signs of antisocial behavior or substance misuse and to prevent consolidation is, therefore, an imperative for society.

The majority of programs involving parents of older adolescents, however, mainly concern adolescents who already have developed and consolidated antisocial behavior. A systematic review from the Cochrane Collaboration has investigated the effects of programs for youth 10–17 years old who had either conduct disorder or involvement in delinquency. The results suggested that the family and parenting interventions had beneficial effects on reducing the time youth spent in institutions, but no effects were found for psychosocial outcomes such as child/adolescent behavior (Woolfenden et al. 2001). In later years, enhanced versions of the SFP have also been successfully implemented with high-risk families with children of different ages. One European example is a quasi-experimental study that indicated positive effects of SFP 12–16 when adapted for use in high-risk families in Ireland (Kumpfer et al. 2012). In the scientific literature, there is, however, insufficient evidence concerning the effectiveness of programs for parents of pre-referred, pre-incarcerated, and undiagnosed adolescents (Leijten et al. 2012). In Sweden as well as internationally, randomized clinical trials of parent programs that aim at reducing at-risk adolescents’ antisocial behavior and substance use are rare.

Against this background, this study was set up to investigate the effects of Comet 12–18 and ParentSteps on measures of antisocial behavior when given under real-world conditions to parents of at-risk adolescents. Though the two programs have some similarities in content (see “Method”), they differ in their theoretical backgrounds. The secondary aim was to test the programs’ effectiveness on a subsample with poorer psychosocial functioning. In both instances, the research question being addressed was whether adolescents in either of the two intervention groups engaged less in antisocial behaviors when followed up than adolescents whose parents had not been given one of the interventions.

## Method

### Participants

Eligible participants were parents/caregivers and their adolescent children aged 12–18 years old who were at risk of consolidating antisocial behavior. When screening parents for participation, antisocial risk behavior in adolescent was defined as the presence of at least one of the following, as indicated by single-item descriptions: delinquent behavior; bullying; repeated conflicts regarding family rules; any use of alcohol, tobacco, and/or drugs; or excessive computer use. The exclusion criteria were the adolescent’s ongoing psychotherapy, treatment for alcohol or drug use, out-of-home placement, and parents’ participation in another parent program. Participation also necessitated living in one of the five participating municipalities (see “Procedure”), and the adolescent had to live at least part-time with the participating parent or caregiver. Because this trial was carried out under real-world conditions, the inclusion criteria used was to reflect the social services’ criteria for inclusion in their regular parent groups. The use of excessive computer use as an inclusion criterion was requested by the social services since many parents described it as being a source of family conflicts and, to some extent, of truancy. Excessive computer use has been associated with health problems, academic failure, and impaired impulse control (Holstein et al. 2014; Moreno et al. 2011), and it has been found to correlate with other forms of addiction such as alcohol, drugs, and gambling (Sung et al. 2013). The study was approved by the Stockholm ethical review board at Karolinska Institutet prior to onset (registration nr 2008-744-31), and the study was registered in Current Controlled Trials (http://www.controlled-trials.com) after participant enrollment (register nr ISRCTN76141538).

### Design

This trial had a simple randomization design with a ratio of 1:1:1 for assignment to Comet 12–18, ParentSteps, or to a six-month wait-list control condition. The randomization sequence was generated by a research assistant drawing one of three folded opaque pieces of paper from a bowl. The paper was then put back in the bowl for the next family to be randomized. The research assistant had not met the parents before randomization, and leaders of the intervention groups were not involved in or aware of the randomization procedure. After randomization, the research assistant provided the group leaders with contact information for the parents who had been assigned to their particular program to enable invitation to the group sessions. After the six-month follow-up, parents in the control group were offered Comet 12–18 or ParentSteps in accordance with their preferences. At the six-month follow-up, 32 parents reported that they themselves or their adolescent child had participated in another intervention targeting the adolescent such as seeing a school counselor or a therapist at a child and adolescent psychiatry unit. These were equally distributed across groups (Comet 12–18 14.1 %, ParentSteps 14.3 %, and control 12.2 %), and they were kept for analysis.

#### Sample Size Estimation

Because no previous study has reported on the effectiveness of parent training for this study group, little information was at hand to anchor the power calculation. Hence, the calculation was based upon the results from a Swedish study on the effectiveness of the Comet 3–11 program (Kling et al. 2010). In that RCT, positive effect sizes for children’s problem behavior reached a Cohen’s d of 1.07 compared to controls using the parents’ ratings on the Parent Practices Interview (PPI) (Webster Stratton 1998; Webster Stratton et al. 2001). As PPI was shown in the mediation analysis by Kling et al. (2010) to causally change the behavior of the child, the PPI construct was chosen for the power calculation. In the present trial, however, it was assumed that the effect size would be substantially lower because parents’ ability to influence their adolescents´ problem behavior diminishes as the adolescent gets older. Considering this, the detectible effect size difference was set to .40, which required a total sample size of 300 subjects with the power set to .80 and the alpha set to .05. With the total actual sample size of 241 parents and 237 adolescents, the detectible effect size difference was .45.

### Procedure

#### Recruitment

Since this trial was carried out in a real-world setting, we sought to emulate the ordinary enrollment procedure of the social services. The usual procedure for parents who seek parent training is to contact the social services that provide these programs to their residents. Parents receive information about the regularly offered programs by ads, pamphlets, or by recommendations. In order to reach the sample size required for this trial, we had to rely not only on recruitment through the social services but also on advertising in local newspapers and on applicable websites. During a one-month period at the beginning of each semester between August 20, 2008, and February 2010, parents were invited by staff at the social services offices and through advertisements to visit a website with study information. The site contained a screening form for eligibility that was based on the inclusion criteria. Eligible families received postal consent forms for both the parent and the adolescent. Those parents and adolescents who returned signed consent forms were randomized as a dyad to the Comet 12–18, ParentSteps, or control group. The majority of parents were recruited to the trial by advertising. The proportions who were recruited by the social services were 18 % in Comet 12–18, 27 % in ParentSteps, and 20 % in the control group. There were no significant group differences in this respect (Comet 12–18/control z = − 0.579, *p* = .56; ParentSteps/control z = 1.77, *p* = .08). Due to the relatively slow influx of participating families, we invited four more municipalities in Stockholm County, Sweden, to participate after the first wave of inclusion, and all four consented. We also added a fourth recruiting wave to the three that were initially planned for. This enlargement—four additional municipalities and one additional recruitment wave—eventually comprised four recruitment waves in the municipalities of Huddinge, Solna, Sundbyberg, Nacka, and 12 of the 14 districts of the city of Stockholm. The selection of municipalities was based on population size, the availability of ParentSteps or Comet 12–18 by the social services, and geographic proximity in order to minimize commuting distance for the participating parents. At the social services, each head of division decided if their parent group leader team would participate, and the contracted group leaders where recruited by an e-mail invitation sent to all certified group leaders in Stockholm County.

#### Measurements

The baseline assessment was conducted prior to the start of the intervention. The research assistant administered the baseline questionnaires to parents in the intervention groups at the first parent group meeting. Postal questionnaires were used for parents in the control group and for subsequent follow-ups six months after baseline in all groups. The majority of adolescents (79 %) answered e-mail–administrated web questionnaires. All participants were ensured confidentiality of self-reported information. Though different modes of assessments, such as self-report and interviewer assessments, might bias the responses (Bowling 2005; Tourangeau and Smith 1996), self-administrated questionnaires are considered to increase respondent openness concerning sensitive questions (Tourangeau and Smith 1996) and to decrease desirability distortion (Hood et al. 2012). The greatest differences seem to be between mode, not within mode (Bowling 2005), and it has been found that the impact of the presence of others is not significantly different from the absence of others when responding to questionnaires (Hood et al. 2012). The first parent group in the first inclusion wave filled out their baseline measurement on September 25, 2008, and the last follow-up for the fourth group who entered the study in spring of 2010 took place on October 19, 2010. The follow-up for each group was planned to occur six months after baseline as reported to the trial registration. The span of response times on the follow-up questionnaire ranged from 1 to 63 days with a mean of 9 days, and thus the follow-up deviated only slightly from the planned date.

### Interventions

#### General Preconditions

The parent programs in this trial were run under real-world conditions by the social services. Eighty-five percent of the parent group sessions were held as part of the social services’ routine work. The rest were held by contracted group leaders at social services facilities or in rented meeting rooms. All partaking group leaders were social workers who were active within the social services either in family or youth units. They were all trained and certified program leaders who had led at least one parent group prior to this study. In order to minimize dropouts, the group sessions had to be carried out within one semester. In the Swedish school system, there are two semesters that start in the middle of August or January, respectively. This necessitated that in less than a month the participants had to respond to the study invitation, apply, consent, be randomized, be allocated to a suitable parent group, and answer the baseline questionnaire prior to the start of the parent group intervention. As shown in Table 1, the number of conducted parent groups differed between the two programs because of the social services’ limited capacity for including trial parents in their regular Comet 12–18 group. This was due to the social services’ obligation to provide parental support to their residents. In some groups there were only two trial parents, while in two groups the whole group comprised parents participating in the trial. In the ParentSteps, almost all parents in each group were participating in the trial.

**Table 1:** Program comparisons

	Comet 12–18	ParentSteps
*Training of group leaders*		
Education/training days	6 + 2 boosters	1
Tutoring while carrying out the first group session	Yes	No
*Program structure*		
Number of weekly parent sessions	9 + 1 optional booster	6
Duration of each session, hours	2–2.5	1.5–2
Recommended group size, parents	6–8	8–12
*Trial characteristics*		
Number of group leaders in the trial	31	13
Number of conducted parent group sessions	30	12
Sessions held within social services routine care	28	8
Sessions held by contracted group leaders	2	4

Because the social services were obliged to deliver the interventions under study to their clients within their ordinary practice, only a limited number of study participants were allowed to enter each intervention group. Due to the smaller recommended group size of Comet 12–18, this limitation was more pronounced and led to more sessions being held than in the ParentSteps program

#### Program Comparisons

Both Comet 12–18 and ParentSteps are manualized and executed as parent group interventions led by two certified leaders. As shown in Table 1, there are differences between the two programs in the amount of training required to become a leader as well as differences in program duration and structure. For example, more training is required for leaders in Comet 12–18 than in ParentSteps (six days + two boosters for Comet 12–18 vs. one day for ParentSteps). Comet 12–18 also has a longer duration with nine group sessions (+one optional booster) compared to six sessions for ParentSteps, and it has a smaller recommended group size. Both programs use video vignettes in each session to illustrate common parent-adolescent interactions, and these serve as a basis for discussions. As further described below, the video films also provide the basic frame for the ParentSteps sessions while they play a less prominent role in Comet 12–18.

#### Comet 12–18

Like Comet 3–11 for younger children (Kling et al. 2010; Kling and Sundell 2006), Comet 12–18 rests theoretically on operant learning and social learning principles (Forster and Livheim 2009). The overall aim of the program is to help parents to develop and enhance their skills and self-efficacy for parenting, thereby preventing consolidation of antisocial behaviors in their adolescents. Principle program components are rehearsals of the use of reinforcement principles (e.g. encouragement and praise and ignoring minor problems) through role-play and home-assignments where parents practice and develop the principles in their daily lives. Parents are instructed to keep a diary to document their interactions with their adolescent and home assignments are followed-up in subsequent sessions. Video vignettes are used in each session to enhance learning. The first vignette shows a common, problematic parent-adolescent situation that is used for discussion in terms of reinforcement principles and possible solutions. The second vignette illustrates how the same problematic situation can be dealt with more constructively. Examples of themes covered during the nine group sessions include taking initiatives for spending time together with the adolescent, dealing with rejection, basic interactional (behavioral) analysis, positive communication and encouragement, problem solving, and rules and consequences. For information on program intensity and duration, see Table 1.

#### ParentSteps

Like the SFP 10–14 (Kumpfer et al. 1996) and its Swedish adaptation Step-by-Step (Skärstrand et al. 2008), the ParentSteps intervention (Larsson et al. 2009) is theoretically based on the Resilience Model (Richardson et al. 1990) and the Social Ecology Model of Adolescent Substance Use (Kumpfer and Turner 1990). The program focuses primarily on the prevention of substance use but it also focuses on other types of antisocial behavior, and it seeks to strengthen protective factors and to reducing known risk factors in parents and adolescents. Family attachment/bonding, parental supervision, and communication of positive family values and norms are viewed as the main protective factors (Kumpfer et al. 2003). For example, “spending-time-together” aims at strengthening the relation between the parent and the adolescent, which in turn is assumed to prevent adolescent problem behavior (Kumpfer et al. 1996). Unlike the SFP 10–14 that includes parent, youth, and joint family sessions, the ParentSteps is unidimensional and addresses parents only. Further modifications in ParentSteps compared to the SFP 10–14 include the reduction from seven parent sessions and four boosters were reduced to six sessions, the omission of the element “small penalties for small problems” (and vice versa), changing “I” statements to “talk-and-listen-communication”, and the addition of a section on tobacco use. One program goal of ParentSteps is to increase parents’ understanding of developmental characteristics of adolescents and to improve their skills in dealing effectively with their adolescents in everyday interactions. This includes, for example, encouraging parents to set appropriate rules with reasonable consequences and to communicate clear expectations regarding substance use and other antisocial behavior. ParentSteps is conveyed and practiced by means of video vignettes, group discussions, and home assignments. The themes for the six sessions and home assignments are Love and limits; Encouragement and consequences; Risks and protection; Stress, fights and different points of view; Youth, parents and alcohol; and Youth, parents and drugs. ParentSteps has a highly structured format, and the video film for each session also provides the time-points for the starting and ending of group discussions and the assignments for that session (Larsson et al. 2009). Thus, the group leaders have limited opportunities to depart from the schedule. For information on program intensity and duration, see Table 1.

### Measures

*Child Behavior Checklist and Youth Self*-*Report (CBCL and YSR).* Achenbach’s CBCL and YSR were used to assess parents’ and adolescents’ ratings of the adolescents’ antisocial and problem behaviors during the last six months. Items are rated on a 3-point scale of 0 (never/seldom), 1 (sometimes), or 2 (often/always). The total problem score (range 0–210 points) and the externalizing behavior broadband syndrome scale, which measures rule breaking and aggressive behavior (range 0–64 points), were used in this study. Higher scores indicate more problem behavior (Achenbach and Rescorla 2001). In the manual for the CBCL and YSR, Cronbach’s α for the total scores are .97 (CBCL) and .95 (YSR), and for the externalizing behavior scores the α value are .94 (CBCL) and .90 (YSR) (Achenbach and Rescorla 2001). In this study, the Cronbach’s α was .92 for CBCL and .94 for YSR, and for the externalizing scale α values were .87 (CBCL) and .89 (YSR).

*Self*-*Reported Delinquency (SRD)*. The total scale score (excluding the subscale hard drug use, which was measured with DUDIT) was used to measure overt and covert behavior that taps violence, general delinquency, and status offenses. Adolescents were asked how many times they committed any of the 40 behaviors on the list during the last six months, and they could answer from “zero” to “nine times or more”. The total score was 0–360 points (Elliot and Ageton 1980). Cronbach’s α for this sample was .92.

*Alcohol Use Disorder Identification Test (AUDIT).* AUDIT was used to measure self-reported alcohol consumption and related problems. The instrument consists of ten items with a total score of 0–40 points. Cut-off points are set to ≥8 for men and ≥6 for women to define risky alcohol use. The instrument is not validated for adolescents aged 12–18 years (Kallmen et al. 2011). Cronbach’s α on the total scale score was .86.

*Drug Use Disorder Identification Test (DUDIT).* DUDIT measures self-reported illicit drug use on 11 items with a total scale score of 0–44 points. No cut-off points are validated for adolescents. For analysis, the follow-up values were dichotomized with 0 = not used during the last six months and 1 = those who had ever used illicit drugs during the last six months (Berman et al. 2005). Cronbach’s α on the total scale score was .92.

*Youth*-*Outcome Questionnaire Self*-*Report (Y*-*OQ*^*®*^*and Y*-*OQ*^*®*^*SR).* Parent and adolescent questionnaires, the Y-OQ^*®*^ and Y-OQ^*®*^SR, respectively, rate adolescents’ psychosocial distress, functioning problems, and behavior change. Sixty-four items with six subscales are rated on a 5-point scale (0 = never, 1 = rarely, 2 = sometimes, 3 = frequently, 4 = almost always), including eight reversed items that measure healthy behaviors (with a range of scores from −2 to 2). The total scale score ranges from −16 to 240 points, and higher scores indicate greater psychosocial dysfunction. The cut-off score of ≥46 was used to define a subsample with clinical levels of psychosocial dysfunction (Burlingame et al. 2005). The Y-OQ^®^-SR total score has demonstrated high internal consistency with a Cronbach’s α of .95 (Ridge et al. 2009), and α of .95 also for the Y-OQ^®^ (Burlingame et al. 2005). Cronbach’s α values in this study were .93 (Y-OQ^®^-SR) and .94 (Y-OQ^®^).

#### Program Implementation Measures

*Dosage* To assess to what extent parents participated in parent group sessions, the group leaders were asked to keep an attendance list. The group leaders in ParentSteps updated the list at every group session, while the Comet 12–18 leaders updated it at every second session.

*Program fidelity* All group leaders self-rated to what degree the program manual and all single session sections were fulfilled, and were measured with questionnaires after the sessions by both group leaders individually. Furthermore, to ensure fidelity only active social workers who were already certified group leaders participated in the study.

### Data Analysis

In 40 of the participating families, both parents entered the trial, and in two families three parents entered the trial. Since only independent respondents’ ratings should be included in the analysis, and because the vast majority of all participating parents were mothers, we only used the mothers’ ratings for the analysis of parents’ data (Moretti and Obsuth 2009). A chi-square test and one-way Analysis of Variance (ANOVA) were used to test for group differences in baseline characteristics. *T* tests were conducted to test the differences in the outcome measures between those who were lost to follow-up and those who were not lost. Analyses of normally distributed outcome data were performed with the General Linear Model (GLM) repeated-measures ANOVA, while the skewed data on the SRD was first log-transformed. Gender and age were added to the ANOVA models as secondary explanatory factors. Severely skewed data were analyzed by the Kruskal–Wallis *H* test for independent samples (AUDIT) and by binary logistic regression after dichotomization (DUDIT). Cohen’s *d* for effect size was calculated for all outcome variables except for AUDIT and DUDIT. Cohen’s operational definitions of effect sizes are ≅0.20 (small, negligible practical importance), ≅0.50 (medium, moderate practical importance), and ≅0.80 (large, crucial practical importance) (Cohen 1987). In addition to the main analyses of outcomes for the complete groups, two kinds of subgroup analyses were performed. One involved subsamples with poorer functioning at baseline that were identified with the Y-OQ cut-off score for clinical levels of psychosocial dysfunction (≥46 points) and with gender-specific AUDIT cut-off scores for (young adult) risk drinking (≥6 for girls and ≥8 for boys). The other subgroup analyses omitted 18-year olds and included only adolescents 12–17 years old in order to improve the comparability with other studies. Lastly, in the Discussion we compare mean values of YSR in a Swedish normal population of adolescents and this study’s sample with Welch’s *t* test for unequal sample sizes in order to demonstrate that we pinpointed an at-risk sample of adolescents.

### Intent-to-Treat and Missing Data

We used Intent-to-Treat (ITT) analysis, in which all participants who were measured at baseline were included in the analyses even if they were dropouts from treatment. ITT analysis was used to analyze the effect of the interventions because they were given in real-world practice. Under such real-world conditions, participants might drop out from treatment and the trial to a larger extent than during more rigorous clinical trials, which can lead to complications when interpreting the results. The ITT approach reduces the risk that the results are biased in favor of treatment. The risk of over-estimating program effects is lower with ITT compared to when only treatment and study completers are analyzed (Keene 2011). This approach entails an estimation of the non-completers’ missing data. The internal non-response rate in the data was low (0.4–3.5 % on single items). Little’s MCAR (Missing Completely at Random) test revealed that the values were missing at random (Donders et al. 2006; SPSSInc 2010), and the missing values were handled with the estimation maximization algorithm single-imputation method. In the rare case where individuals were lost to follow-up (parents 1.2 %, adolescents 4.2 %), the Last Observation Carried Forward procedure was performed.

## Results

**Fig. 1:**
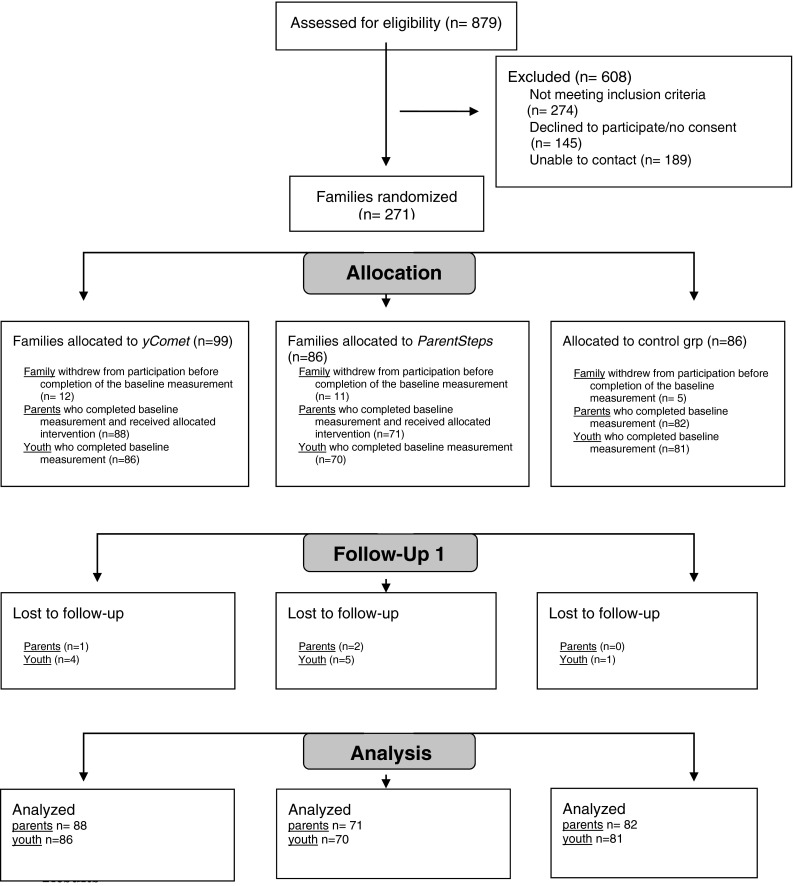
CONSORT 2010 flow diagram of participants. Flow chart of recruitment and dropout following the Consort Statement 2010 Flow Diagram

Figure 1 shows the flow of participants through the trial. Participants who did not answer the baseline measurement were omitted from the study even though they had been randomized. The procedure to randomize the participants prior to the baseline assessment had to be overlooked in favor to the short time span of recruitment, randomization, measurement, and group participation as described above. Among the 241 parents and 237 adolescents who participated in the baseline assessment, only three parents (1.2 %) and ten adolescents (4.2 %) were lost to follow-up. There was no significant difference between measurement completers and those lost to follow-up regarding externalizing behavior (t = − 1.39, *df* = 237, *p* = .17), and there was no significant difference between conditions with regard to attrition rates (χ^2^ (2) = 2.99, *p* = .22).

Table 2 shows the baseline characteristics of the participants by group. There were significantly fewer girls in the Comet 12–18 group compared to the control group. No other differences were observed between groups. Regarding the outcome measures, we found no significant differences between the groups, with exception of the DUDIT scores (Table 5). At baseline, 70 % of the adolescents reported ever having used alcohol, and 53 % of the girls and 23 % of the boys scored above the cut-off defining risky alcohol use on the AUDIT scale. The vast majority (95.4 %) of the adolescents reported having engaged in any delinquent behavior during the last six months that preceded the baseline measurement.

**Table 2:** Baseline characteristics of the study sample

Variable	Comet 12–18	ParentSteps	Controls	Test statistic	*p* value
Adolescents, n	86	70	81		
Parents, n	88	71	82		
Girls	38.4	45.7	58.0	χ^2^ (2, N = 237) = 6.56	.04
Mean age (SD)	14.6 (1.67)	14.5 (1.63)	14.7 (1.89)	F (2, N = 237) = 0.38	.69
Participating mothers/stepmothers	92.0	95.8	93.9	χ^2^ (2, N = 241) = 7.39	.27
Participating fathers/stepfathers	8.0	4.2	6.1	χ^2^ (2, N = 241) = 7.39	.27
More than one parent in the trial	18.2	15.5	17.1	χ^2^ (2, N = 241) = 0.20	.90
Foreign-born mother	19.8	20.0	20.9	χ^2^ (2, N = 237) = 0.9	.92
Single-parent family	43.7	50.7	41.5	χ^2^ (2, N = 240) = 1.41	.49
Parent has university degree	28.4	17.1	28.0	χ^2^ (2, N = 240) = 3.26	.20
Parent is employed	86.4	84.5	84.1	χ^2^ (2, N = 241) = 0.19	.91

Values are percentages or mean values and SD

Of the six sessions in total, parents in the ParentSteps attended a mean of 4.7 sessions (SD = 1.44). Of the nine Comet 12–18 sessions in total, only every second session was assessed and the mean attendance for 4.5 sessions was 3.6 (SD = 1.07). With dropout defined as absence during the two last sessions, the dropout rate in the ParentSteps was 14.1 %. It was impossible to estimate the dropout rate in Comet 12–18 due to the lack of data from the two last sessions. Only social workers who were certified as program deliverers and were active as group leaders were involved in the trial. Comet 12–18 leaders reported that 78 % of the 73 sections in the manual were fulfilled “to a full extent”, 17 % “to a greater extent”, 3 % “to at least half”, 1 % “to a lesser extent”, and 1 % “not at all”. ParentSteps leaders’ self-assessments showed that 83 % had fulfilled the manual sections “to a full extent”, 13 % “to a greater extent”, 2 % “to at least half”, 1 % “to a lesser extent”, and 1 % “not at all”.

As shown in Table 3, in the parents’ rating on CBCL total score and the externalizing behavior subscale score, all three groups decreased significantly over time, but no significant differences between the groups over time were found. The same patterns can be seen in parents’ ratings on Y-OQ^®^ and when using the cut-off score ≥46 points to define a subsample with clinical levels of psychosocial dysfunction. For parents with adolescents in the age group 12–17 years, the results were similar to the total sample with non-significant group differences for the CBCL (F (2, 221) = 0.80, *p* = .45), externalizing behavior (F (2, 221) = 1.14, *p* = .32), and the Y-OQ^®^, (F (2, 221) = 0.14, *p* = .87). For adolescents’ ratings, a statistically significant improvement in psychosocial functioning over time was observed in the total sample as well as in the clinically defined sub-sample, but again no group-by-time effects were found. There was no effect of time in adolescent’s reports of anti-social behavior for the total or for the clinical sub-sample. For the age group 12–17 years, the results were similar to the total sample, with non-significant differences for the YSR (F (2, 227) = 0.21, *p* = .81), externalizing behavior (F (2, 227) = 0.50, *p* = .61), Y-OQSR^®^ (F (2, 227) = 0.65, *p* = .52), and the SRD (F (2, 227) = 0.31, *p* = .73). Gender and age were added to the ANOVA models but did not account significantly for any within-group variation, and the effect estimates remained basically the same.

**Table 3:** Behavioral and delinquency outcomes—descriptive statistics by group, effects of time and time multiplied by group, and accompanying effect sizes

	Comet 12–18		ParentSteps		Control		Time			Time × group			Effect size (Cohen’s d)	
	T1	T2	T1	T2	T1	T2	*df*	*F*	*P*	*df*	*F*	*p*	Comet 12–18/control	ParentSteps/control
Variable, means and standard deviations (SD)														
*Parent reports*														
CBCL, total score	49.21 (22.04)	28.30 (23.51)	50.31 (26.87)	33.96 (27.27)	47.26 (25.11)	31.59 (22.65)	1, 241	152.14	.00	2, 241	1.40	.25	.22	.03
Y-OQ, total score	51.95 (29.64)	41.44 (29.35)	53.06 (30.17)	43.60 (32.93)	55.39 (31.67)	46.14 (28.73)	1, 241	44.36	.00	2, 241	0.07	.93	.04	.01
Y-OQ total score, clinical sample	72.01 (19.10)	55.34 (24.67)	71.99 (20.94)	59.73 (30.56)	74.22 (26.52)	59.97 (28.20)	1, 144	53.69	.00	2, 144	0.42	.66	.10	−.01
*Youth self*-*reports*														
YSR, total score	45.31 (24.16)	46.00 (28.83)	46.50 (24.42)	45.16 (25.32)	47.22 (24.32)	46.90 (27.90)	1, 237	0.03	.86	2, 237	0.24	.88	−.04	.04
YSR, externalizing behavior	18.27 (10.45)	18.31 (11.35)	17.29 (9.29)	16.59 (10.66)	17.29 (9.12)	17.15 (9.96)	1, 237	0.18	.67	2, 237	0.09	.93	−.02	.06
Y-OQ, total score	44.28 (28.30)	40.210 (30.69)	45.68 (32.65)	42.05 (32.45)	48.12 (30.40)	42.04 (33.97)	1, 237	7.57	.01	2, 237	0.20	.81	−.07	−.08
Y-OQ total score, clinic sample	70.42 (21.80)	60.50 (29.49)	74.70 (23.02)	62.17 (30.10)	73.05 (23.34)	59.65 (33.19)	1, 108	19.70	.00	2, 108	0.216	.85	−15	−.04
SRD, total score	38.94 (46.14)	41.13 (53.36)	29.20 (27.99)	31.49 (40.68)	30.80 (35.40)	28.68 (36.81)	1, 237	0.09	.76	2, 237	0.32	.73	−.10	−.12

*CBCL* Child Behavior Check List (parent version), *YSR* Youth Self-Report (CBCL youth version), *Y-OQ* ^*®*^ *and Y-OQ* ^*®*^ *SR* Youth Outcome Questionnaire (parent and youth self-report versions), Y-OQ^®^ cut-off score ≥46 points on total scale, *SRD* Self-Reported Delinquency. Values are baseline (T1) and 6-month follow-up (T2) mean values and (SD). Analyses by GLM repeated measures ANOVA: effect of time (between T1 and T2), and effects of time (T1 and T2) and group. Cohen’s d is calculated using differences between T1 and T2 and pooled SD in both interventions versus control. Adolescents’ gender and age were added and controlled for in the ANOVA models

As Table 4 shows, the analysis with the Kruskal–Wallis *H* test on the follow-up data indicated that the distributions of the AUDIT scores were similar across groups for the total sample (*p* = .07) and for the subsample with elevated alcohol risk use (*p* = .57 and *p* = .91 for girls and boys, respectively), and no group effect was found. In the younger age group (≤ 17 years old) the Kruskal–Wallis *H* test showed a statistically significant difference in the between-group distribution at follow-up (χ^2^ (2) = 6.033, *p* = .049) with a mean rank of m = 131.1 for Comet 12–18, m = 105.1 for ParentSteps, and m = 118.11 for controls. However, in the post hoc test with the significance level adjusted for multiple testing, all pairwise comparisons indicated non-significant differences between distributions.

**Table 4:** Adolescents’ self-reported alcohol use and test of group differences at follow-up

Variable	Comet 12–18		ParentSteps		Control		*p* value
	T1	T2	T1	T2	T1	T2	
	m (SD)	m (SD)	m (SD)	m (SD)	m (SD)	m (SD)	
AUDIT, total score^a^	6.20 (6.31)	7.59 (7.60)	5.59 (7.43)	5.10 (6.38)	6.04 (6.27)	6.26 (6.79)	.06
AUDIT, risky use among girls^b^	11.47 (6.22)	11.21 (7.274	14.80 (7.34)	12.73 (6.73)	11.54 (4.02)	11.85 (6.16)	.24
AUDIT, risky use among boys^c^	12.56 (4.95)	13.81 (9.08)	12.90 (3.66)	10.30 (4.22)	14.11 (4.68)	10.33 (5.70)	.51

Values are baseline (T1) and 6-month follow-up (T2) mean (m) values and SD for alcohol use by group. The *p* value refers to the test of group differences at follow-up using the Kruskal–Wallis *H* test

^a^n = 237 (Comet 12–18 n = 86; ParentSteps n = 70; Control n = 81)

^b^n = 61 (risky use among girls: AUDIT score ≥6. Comet 12–18 n = 19; ParentSteps n = 15; Control n = 26)

^c^n = 36 (risky use among boys: AUDIT score ≥8. Comet 12–18 n = 16; ParentSteps n = 10; Control n = 9)

Binary logistic regression analysis of DUDIT scores (Table 5) showed that adolescents whose parents participated in any intervention had more than threefold significantly elevated odds of using illicit drugs at follow-up. The 95 % confidence interval (CI) of the effect estimates was [1.24, 10.72], and in the younger sub-group the 95 % CI was [1.12, 12.33]. Table 5 also shows that the proportion of drug users had increased in both intervention groups during follow-up, while it decreased from 21 to 11 % in the control group.

**Table 5:** Adolescents’ self-reported illicit drug use and test of group differences at follow-up

	Any drug use (%)	Odds ratio (95 % CI)
Comet 12–18, n = 85		
T1	21.2	3.52 (1.23–10.10)*
T2	25.9	
ParentSteps, n = 70		
T1	7.1**	3.23 (1.06–9.08)*
T2	17.1	
Control, n = 81		
T1	21.0	
T2	11.1	

T1 (baseline) and T2 (follow-up) values (percent) for any illicit drug use as measured with DUDIT by condition, and the test of group differences by logistic regression at T1 and T2. The control group is used as the reference category. Significance test of the difference between intervention and controls was carried out with the Chi squared test. The baseline value of the outcome was included in the model

* *p* < .05; ** *p* < .01

## Discussion

This randomized trial aimed to evaluate the effects of two Swedish prevention programs for parents of adolescents who are at risk for antisocial behavior. The prevention programs were provided under real-world conditions by the social services in the greater Stockholm urban area. Our results suggest that neither the Comet 12–18 nor the ParentSteps programs have beneficial effects on adolescents’ externalizing and delinquent behavior or substance use as reported by adolescents and parents in comparison to a six-month wait-list control group. Even though we also analyzed sub-groups with elevated levels of problem behavior assessed with Y-OQ^®^ and risk levels of alcohol use with AUDIT, no significant group effects emerged. Nor were any significant differences detected between the groups in a secondary analysis of the sub-group of adolescents 12–17 years old.

The only significant, and the most salient, finding was that the risk of illicit drug use at follow-up was threefold greater in both intervention groups compared to controls, suggesting that the studied parenting programs might actually have caused harm in this particular outcome. Although harmful effects of interventions are of outmost importance to consider when they appear, we believe there are several reasons to treat this finding with caution. First, this was the result of an increase in drug use in the past six months in the intervention groups between baseline and follow-up and a concurrent decrease in the control group from 21 % to 11 %. Given that drug use among Swedish adolescents in general is known to increase between the ages of 15–18 years (Kallmen et al. 2011), the development in the untreated control group might better be understood as a measurement error. Second, the low occurrence of drug use in the sample makes the estimates uncertain, as displayed by the wide confidence intervals [95 % CI (1.24, 10.72)]. Finally, the theoretical mechanisms by which the two programs might lead to an increase in drug use (and at the same time leaving other outcomes unaffected) are difficult to comprehend.

One possible explanation for the null findings is that the Comet 12–18 and ParentSteps are, in fact, not effective for reducing antisocial behavior in this population of at-risk adolescents. The systematic review from the Cochrane Collaboration by Furlong and colleagues (2012) showed that cognitive behavior therapy-based parent programs such as Comet 12–18 can be effective for improving conduct problems in younger children, but the authors note that the results might not necessarily be generalizable to cognitive-behavioral programs for other age groups (Furlong et al. 2012). Also, results from other systematic reviews have pointed to the value of supporting families and parents early in the children’s lives (Piquero et al. 2009). Though age did not fall out as a significant moderator in their meta-analysis of behavioral parent-training programs, Lundahl et al. (2006) found the highest effect sizes in studies with children up to 5 years old (*d* = 0.44), somewhat lower effect sizes for children 6–10 years (*d* = 0.31), and the lowest effect sizes for children 12 years and older (*d* = 0.27) (Lundahl et al. 2006). The task of developing new interaction patterns when the child has reached the adolescent years might be more difficult, and might be, like Hindelang et al. (2001) state, “… much like closing the barn door after the horse has left” (p. 82). Compared to younger children, adolescents spend more time outside the home and the influence from peers grows relatively stronger. If developing new interaction patterns is more difficult than with younger children, a follow-up period of six months might also be a too short a time to allow effects to be seen in adolescents’ problem levels.

The SFP assumes a developmental perspective, with the family exerting relatively more influence on children and young adolescents than on older adolescents (Molgaard and Spoth 2001). With regard to the ParentSteps program for parents of at-risk adolescents (Larsson et al. 2009), the program was theoretically modeled on the Swedish adaptation (Skärstrand et al. 2008) of the universal SFP 10–14 (Molgaard and Spoth 2001). However, ParentSteps departs substantially from the SFP structure by only including parent sessions (i.e. no youth or family sessions). As briefly reviewed in Kumpfer et al. (2002), the original experimental SFP research found that the combined parenting, youth, and family intervention (which constitutes SFP today) led to significantly greater improvements in parent, child, and family risk factors in the older children and parents than did the stand-alone parenting sessions (DeMarsh and Kumpfer 1985; Kumpfer et al. 2002). When SFP 12–16 was adapted and successfully implemented with Irish high-risk families, the multimodal program structure was left intact (Kumpfer et al. 2012). A related issue is that ParentSteps is a slightly shorter program than the universal SFP 10–14, as it includes six rather than seven sessions. In contrast, SFP 12–16 in the Irish study mentioned above was twice as long as the universal SFP 10–14 version (i.e. 14 sessions), and a quasi-experimental study also suggested positive effects of the intervention (Kumpfer et al. 2012). The modifications made to the ParentSteps have interfered with what has been referred to as the “deep structure” (Resnicow et al. 2000) of the SFP programs, i.e. components that need to be left intact when adapting family skills-training interventions to new contexts. As described by Sundell et al. (2014), examples of such deep structure components include “…program activities that strengthen parental skills in communication and child supervision or monitoring…” (p. 7). Though such skills are also taught in ParentSteps, the youth and family sessions of SFP (or the longer duration of SFP) might bring complementary benefits that perhaps are necessary to enable actual change in adolescent behavior.

The data used in the study were based on self-report measures such as the AUDIT. Thus, over- and under-reporting of alcohol use due to exaggeration and social desirability might have occurred. However, considerable evidence suggests the validity of self-reported substance use among adolescents when compared to other sources of data (Hamilton et al. 2008). Also, when rating adolescents’ personality or problem behavior self-rating is said to be one of the most valid methods (Laidra et al. 2006; Saudino et al. 2004; Zukauskiene et al. 2004). When distributing the questionnaires to the adolescents, the confidentiality when handling the data was emphasized, and previous research suggests that self-reports are generally considered reliable when confidentiality is ensured (Campanelli et al. 1987). Thus, a bias in the study results due to the use of self-report measures should not necessarily be expected. Also, the majority of instruments used in this trial have been shown to be valid and reliable self-report measures of adolescent behavior and to be sensitive to changes in outcome studies (Achenbach and Rescorla 2001; Broberg et al. 2001; Elliot and Ageton 1980; Ridge et al. 2009).

The inclusion criteria for this study were designed to reflect those commonly used by the social services for entering the two programs. Consequently, parents were eligible for inclusion if they reported repeated conflicts with their adolescent regarding family rules or if their adolescent engaged in alcohol, tobacco, or drug use, delinquent behavior, bullying, or excessive computer use. These criteria represent a diverse array of behaviors, some of which are not frequently used as indicators of at-risk status for antisocial behavior. One such criterion was the adolescent’s excessive computer use, which has been found to co-exist with academic failure, problems at home, dysfunctional coping strategies, and problematic interpersonal relations (Milani et al. 2009). Since the prevalence of excessive computer use is quite low—4.4 % in Europeans of all ages—and the comorbidity is quite high (Weinstein et al. 2014), we did not expect a large number of adolescents to enter the study solely due to excessive use of computers. During recruitment, 12 of 879 screened parents reported excessive computer use as the main adolescent problem, but none of these parents completed their application and none participated in our study.

A relevant question is whether the study has managed to reach the group of at-risk adolescents for which the two programs are intended. A comparison with Swedish YSR norms (Broberg et al. 2001) shows that the girls in this sample had significantly higher levels of externalizing behavior than girls 13–18 years old in the general population [M = 20.06, SD = 9.44 vs. M = 13.24, SD = 6.92, respectively; Welch’s *t* (121) = 7.47, *p* < .0001]. Boys in the sample also had significantly higher levels of externalizing problem behavior compared to boys in the general population [M = 15.49, SD = 9.35 vs. M = 13.77, SD = 7.92, respectively; Welch’s *t* (145) = 2.00, *p* = .047]. Concerning the parents’ ratings, the parents in this trial rated their adolescents’ levels of problem behavior significantly higher than parents of 12–16 year olds in the general population (Larsson and Frisk 1999) [Externalizing behavior: M = 19.61, SD = 9.65 vs. M = 5.5, SD = 5.7, respectively; Welch’s *t* (304) = 21.35, *p* < .0001].

With regard to alcohol use, there are no Swedish AUDIT norms available for adolescents, but data are available for young adults 17–27 years old (Kallmen et al. 2011). The drinking levels reported by the adolescent girls in this sample were significantly higher than the levels reported by young women this age in the general population [M = 7.1, SD = 7.16 vs. M = 5.1, SD = 3.63, respectively; Welch’s *t* (172) = 2.46, *p* = .01], while the adolescent boys in this study had significantly lower drinking levels than those reported by young men in the general population [M = 4.96, SD = 5.95 vs. M = 7.8, SD = 6.68, respectively; Welch’s *t* (60) = 2.40, *p* = .02]. Because the alcohol use and proportions of risky drinkers in the general population are higher among young adults than in other age groups (Ramstedt et al. 2010), the AUDIT levels reported here suggest that the girls in this trial belonged to the target group of adolescents at elevated risk while the boys did not.

When comparing the trial population to normal population levels, there is some uncertainty stemming from the fact that the YSR and CBCL norms are approximately 15 years old and that the AUDIT norms are for ages 17–27 years. Nevertheless, the inclusion criteria and the recruitment procedure in this trial might selectively have attracted parents of girls with elevated problem levels in terms of externalizing problem behaviors and alcohol drinking and, for some reason, attracted parents of boys within the normal range for risky alcohol consumption. Though the gender variable did not account significantly for any within-group variation when added to the ANOVA models, the fact that boys did not differ from the general population in terms of risk might have limited the possibilities for the interventions to have an effect on the group as a whole (i.e., due to loss of statistical power).

Regarding gender differences, a Finnish study of 240 pupils in the ninth grade (mean age 15.7 years, SD = 4 months) found that heavy-drinking girls scored higher on both social and psychological problems in comparison to both boys and light-drinking girls (Laukkanen et al. 2001). Alcohol use disorder in late adolescence has been found to be associated with being exposed to family conflicts in childhood, and in a third of the cases the association was explained by high levels of externalizing behavior (Skeer et al. 2009). Skeer et al. (2011) studied gender differences in the previously mentioned sample and found that family conflict was associated with substance-use disorder that was partly explained by conduct problems, but this association was only among girls. These results are very interesting and bring intriguing perspectives on young females’ alcohol use and associated risk factors. The aim of the present trial, however, was not to study gender’s association with the outcome measures, but it is possible that this trial’s female adolescents with high scores on the AUDIT also experienced more problem behavior and family conflicts than did boys.

Finally, while significant reductions over time were seen in parent´s ratings (but not between groups), adolescent’s self-reported problem levels did not change significantly between measurements for the majority of outcomes. The reasons for these discrepancies are not clear, but the study recruited parents who were concerned about their children and their behavior, while the adolescents themselves might have regarded their behaviors as less problematic. This might have caused parent’s ratings to regress towards the population mean at re-assessment (Kazdin 2003) leaving the adolescents’ reports unchanged.

Though this study has strengths such as a randomized design carried out in real-world settings and an exceptionally low attrition rate, it carries two limitations that both have implications for the interpretation of the findings. The first is that measures of program implementation in terms of participant responsiveness were not included, i.e., there was a lack of more rigorous measure of dosage, and the measure of program integrity relied on the group-leaders’ self-reports. Our results suggest that neither of the two programs were effective when given under real-world conditions by certified group leaders, which is an important result in itself. However, we cannot present sufficient results on dose–response effects or reliably state the degree to which the programs were given as intended. The second limitation is that measurement of the assumed mediators would have provided valuable information on whether the programs led to improvements in, for example, parent-adolescent communication and bonding and more effective rule setting. Such information would have enabled mediation analyses and facilitated the interpretation of the results.
